# Development and Validation of LC-MS/MS Method for the Determination of 1-Methyl-4-Nitrosopiperazine (MNP) in Multicomponent Products with Rifampicin—Analytical Challenges and Degradation Studies

**DOI:** 10.3390/molecules28217405

**Published:** 2023-11-03

**Authors:** Anna B. Witkowska, Aleksandra Wołczyńska, Agnieszka Lis-Cieplak, Elżbieta U. Stolarczyk

**Affiliations:** 1 Spectrometric Methods Department, National Medicines Institute, 30/34 Chełmska, 00-725 Warsaw, Poland; anna.witkowska@wum.edu.pl (A.B.W.); a.wolczynska@nil.gov.pl (A.W.); a.lis@nil.gov.pl (A.L.-C.); 2 Department of Drug Chemistry, Doctoral School, Medical University of Warsaw, 61 Żwirki i Wigury, 02-091 Warsaw, Poland

**Keywords:** genotoxic nitrosamines, trace level analysis, impurity profile, tuberculosis (TB) drug, stress study, degradation mechanism

## Abstract

Rifampicin is an essential medicine for treating and preventing tuberculosis (TB). TB is a life-threatening infectious disease and its prevention and treatment are public health imperatives. In the time of a global crisis of nitrosamine contamination of medicinal products, patient safety and a reduction in the number of drug recalls at the same time are crucial. In this work, the LC-MS/MS method was developed for the determination of the 1-methyl-4-nitrosospiperazine (MNP), a genotoxic nitrosamine impurity in various products containing rifampicin at a 5.0 ppm limit level according to Food and Drug Administration (FDA). Extraction with neutralization was necessary due to the matrix and solvent effect associated with the complexity of the rifampicin product. The developed method was validated in accordance with regulatory guidelines. Specificity, accuracy, precision, limit of detection, and limit of quantification parameters were evaluated. The recovery of the MNP was 100.38 ± 3.24% and the intermediate precision was 2.52%. The contamination of MNP in Rifampicin originates in the manufacturing process of the drug. Furthermore, the results of the forced degradation experiments show that the formation of MNP is possible by two mechanisms: through degradation of rifampicin and the oxidation of 1-amino-4-methyl-piperazine. This article points out that it is necessary to monitor and describe degradation products and the mechanism of degradation of potentially affected active pharmaceutical ingredient (API) with respect to the formation of nitrosamines during stress testing, as it was done in the following work for rifampicin in multicomponent products.

## 1. Introduction

**Figure 1:**
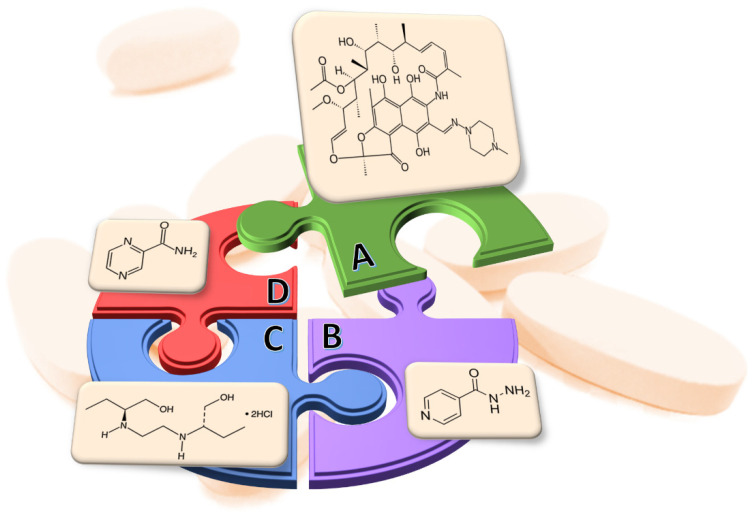
Structures of API included in rifampicin-containing products: (**A**) rifampicin; (**B**) isoniazid; (**C**) ethambutol hydrochloride; (**D**) pyrazinamide.

Rifampicin (Figure 1A), also referred to as rifampin, belongs to rifamycins—an antimicrobial class of drugs. It is used for the treatment of numerous Gram-positive cocci, including *Mycobacterium tuberculosis*, *Clostridium difficile* and selected Gram-negative bacteria: *Neisseria meningitides*, *Neisseria gonorrhoeae* and *Hemophilus influenzae* infections [1,2,3]. FDA approved rifampicin as the first rifamycin antibiotic for treating tuberculosis [4]. Other medical indications for rifampicin include leprosy and less common diseases such as osteomyelitis, endocarditis, brain abscess, meningitis, and implant infections [3]. The advantage of rifampicin administration in the treatment of tuberculosis is that it has the potential to reduce the duration of therapy and has a broad antimicrobial effect [2]. High concentration of rifampicin is also well-tolerated by patients due to the rapid metabolism in the liver. This medicine is available for oral and intravenous administration. It is also highly lipid-soluble, rapidly absorbed, and distributed throughout the body [3]. For active tuberculosis it is often used together with isoniazid (Figure 1B), ethambutol hydrochloride (Figure 1C), and pyrazinamide (Figure 1D). Rifampicin is an extremely important medicine due to the small number of other active substances available for the treatment of tuberculosis. In particular, in times of global crisis of nitrosamine contamination of medicinal products, patient safety and a reduction in the number of drug recalls at the same time are crucial [5]. For that purpose, the content of the impurities, especially those of very probable cancerogenic properties, should be limited as much as possible. Therefore, the drugs should be well tested and their impurity profile should be known. The International Council for Harmonisation of Technical Requirements for Pharmaceuticals for Human Use (ICH) M7 guideline indicates that aflatoxins, *N*-nitrosamines (NAs), and alkyl-azo compounds are highly carcinogenic and genotoxic impurities. Hence, they are grouped as a cohort of concern [6]. *N*-nitrosamines are considered potent genotoxic agents based primarily on data from animal models. This is due to the mutagenic effect which can lead to cancer development. *N*-nitrosamines can arise from several sources: may be present in the active pharmaceutical ingredients (APIs) used in final pharmaceutical products (FPPs) fabrication, may be formed during drug synthesis processes, accidentally introduced due to cross-contamination, solvents recovery procedures, or from degradation of drug substances during storage [5,7,8,9,10]. It is necessary to note that the formation of NAs impurities can be specific for a certain product or nonspecific. It is important to perform a meticulous analysis of risk evaluations of nitrosamines formation.

In August 2020, the United States Food and Drug Administration (FDA) released information about 1-methyl-4-nitrosopiperazine (MNP) contamination in rifampicin. MNP is an intermediate for the synthesis of two substrates, i.e., 1-methylpiperazine and 1-amino-4-methylpiperazine, which are used for the synthesis of drugs [11]. During the synthesis of 1-amino-4-metylopiperazine, MNP is reduced with zinc powder in acetic acid or with hydrogen using a palladium catalyst [12]. Moreover, 1-amino-4-methylpiperazine constitutes one of the starting materials for the synthesis of rifampin. This amine may be the source of the MNP appearing in the final product of the drug. Is the synthesis route the only source of MNP in rifampicin? Well, no. It was reported that the MNP compound arises from the decomposition of the sample as a result of high temperature [13]. MNP belongs to the nitrosamine class of compounds, some of which are classified as probable (IARC Group 2A) or possible human carcinogens (IARC Group 2B) [5]. However, the carcinogenicity of MNP contrasts sharply with the strong carcinogenicity of dinitrosopiperazine, for example. This is difficult to explain since the alpha hydrogen atoms are highly reactive, and exchange readily with deuterium in basified D_2_O [14]. On the other hand, mononitrosopiperazines are mutagenic in the host-mediated assay, in which, indeed, 1-nitroso-4-methylpiperazine is much more active than dinitrosopiperazine [15]. The explanation might lie in the highly polar nature of the mononitrosopiperazines, both of which are strong bases and, therefore, possibly unable to enter cells. But nitrosonornicotine, which is also a strong base, is an effective carcinogen in rats [16]. It must be concluded that conformational factors, some substituents can greatly increase carcinogenic activity in a nitrosamine molecule, other substituents can reduce carcinogenic activity greatly, or even eliminate it, and polar substituents seem to be particularly effective in accomplishing the latter [17]. Ideally, MNP as a nitrosamine impurity should not be present in medicines. But where the presence of MNP cannot be eliminated entirely, they should be controlled below a level where human cancer risk associated with the exposure is negligible. Manufacturers of rifampicin should expeditiously implement remediation plans to reduce the presence of MNP impurity and should test all batches before drug release to ensure the interim limit of 5 ppm [18]. However, limits of 0.16 ppm (FDA) or 0.67 ppm (European Medicines Agency) are currently under consideration [13,17,19]. Highly sensitive analytical methodologies are necessary not only because of impurity trace determination at the ppb level but also because of the significant and different effects of the matrix on the analytes being determined. Liquid chromatography–tandem mass spectrometry, both LC-HRMS [20,21,22] and LC-MS/MS and gas chromatography GC-MS have been widely used for the analysis of NAs [23,24]. By itself, LC or GC with MS detection is very sensitive. However, to develop a sufficiently sensitive and selective analytical method it is necessary to develop a proper sample preparation protocol, which can include solid phase extraction, preparative column chromatography, recrystallization, phase exchanges, and fractional distillation [25,26,27]. The sample treatment not only enables analyte preconcentration but also removes components of the matrix that may interfere with the analysis protocol. Five methods for the determination of MNP in medicinal products have been described in the literature using LC-MS/MS, SFC-MS/MS, and GC-MS/MS, and various extraction methods, including SALLE and LLE [28,29,30,31,32]. Gas chromatography coupled with mass spectrometry was used for the determination of MNP in cetirizine, amlodipine/bisoprolol, sunitinib, olmesartan and cilostazol [28,31]. Rifampicin, according to the literature and our experimental data (on GC-MS/MS), decomposes at elevated temperatures to MNP, so it was impossible to use the GC-MS/MS method for determination of MNP in rifampicin products. MNP in single-ingredient rifampicin capsules has been analyzed by LC-HRMS and LC-MS/MS [13,22]. The HPLC-MS/MS-based method for the detection of MNP in rifampicin capsules was developed by Tao et al. [13]. This is the de facto method of determining MNP in the API since rifampicin spilled out of the capsule constitutes the examined sample. The 2D-LC-UHPLC-MS/MS method developed by de Souza et al. for monitoring *N*-nitrosamines in rifampicin is also exclusively applicable to APIs [29]. Additionally, Tao et al. suggest that MNP is a thermal degradation product of rifampicin which occurs during forced degradation experiments [13]. Their results suggest that temperature during production and sample storage should be strictly controlled. To our knowledge, methods for determining MNP with stress studies in complex multi-component matrices with rifampicin have not been yet reported in the literature.

Our method, developed and described within this article, can be applied to more complex matrices than rifampicin alone. Pharmaceutical complex formulations, for which sample preparation is important, namely tablets containing rifampicin, isoniazid, pyrazinamide, and ethambutol hydrochloride may be analyzed. Thus, it also provides an answer to the question: Should the risk of presence and determination of 1-methyl-4-nitrosopiperazine (MNP) be considered only for the rifampicin finished products? The development of the method was a significant challenge due to the notable matrix effect and solvent effect that was observed in particular for the four-component product. Additionally, we observed that in the case of stress testing on rifampicin-containing tablets, it is not only the temperature that elevates the level of MNP. Therefore, we have applied the newly developed analytical method to study the stability of rifampicin-containing products in terms of the formation of the nitrosamine MNP the source of which may be in the degradation main studied API. From our perspective it is necessary to monitor and describe the degradation products and mechanism degradation of potentially affected API concerning nitrosamines formed during stress testing, what we have done for rifampicin in multicomponent products.

## 2. Results

### 2.1. Method Development 

Due to the high polarity and low molecular weight of MNP, developing an LC-MS methodology was a challenging task. To develop a method for the quantitative analysis of MNP in multicomponent finished products with rifampicin, several analyses were carried out to choose the best column configuration for minimum analysis time with high efficiency and resolution. LC conditions were optimized by screening such columns as Acquity CSH, Kinetex F5, Cosmosil 2.5 HILIC, and Hypersil GOLD Phenyl. The best peak shape of the analyte and shortest analysis time were obtained for the column Purospher^®^ STAR Phenyl.

The important part related to the development of the LC-MS/MS method also concerned the design of a suitable solvent gradient that would allow the MS valve to cut off active substances and placebo for determination of MNP at very low levels without the problems associated with ionization suppression.

Optimization of MS/MS condition was made using Agilent Optimizer (B.07.01) MRM not only for MNP but also for 1-amino-4-methylpiperazine and 1-methylpiperazine compounds were determined for selectivity and stress studies. MNP-d4 was selected as the internal standard.

The most difficult part of optimizing the MNP determination method was to develop a suitable sample preparation method to efficiently extract the MNP into the solution. The first developed method for preparation of the sample for component products 1, 2, and 3 with rifampicin was extraction into 5 mL of methanol, followed by centrifugation and filtration of the supernatant. This method did not work for 4-component products with rifampicin, as a broad peak with low intensity from MNP was observed. The investigation of the pH of multi-component products with rifampicin in aqueous solution showed that one-component products have a pH of 6.45, 2-component products have a pH of 6.63, and 3-component products have a pH of 7.54, while 4-component products have a pH of 4.63. The lower pH of the 4-component solution of the rifampicin product originates from the presence of ethambutol hydrochloride in the product, which caused a matrix effect and solvent effect. The matrix effect was observed in the chromatogram as a loss response for the target analyte and internal standard. Therefore, a second extraction method was developed for the 4-component product with rifampicin to a solution of methanol: water (1:1, *v*/*v*) with the addition of a base (NaOH). The ratio of water to methanol was optimized to obtain the best possible extraction. The developed method was validated, and its results were compared with the method of extraction into methanol. The results of both methods were presented in Table S1.

### 2.2. Validation Results

The specificity of the method was verified as described in Section 4.6, no interference was observed in the retention time of MNP, the standard added to the sample did not cause peaks splitting and the matrix did not affect the result (Figures S1–S12). Figure 2 and Figure 3 show chromatograms of standard solution for MNP and MNP-d4, respectively. 

Results for range, linearity, accuracy, limit of detection (LOD), and limit of quantification (LOQ) were summarized in Table 1. The linearity of the method was evaluated in the concentration range of 0.51–48.62 ng/mL for MNP, and the coefficients of determination R^2^ for MNP were ≥0.999, clearly indicating very good detection responses. The method range was established from the LOQ to up to 280% of the specification limit due to the high content of MNP in some of the samples tested. The recoveries for MNP were in the range of 100.38 ± 3.24% with RSD = 8.17%.

**Figure 4:**
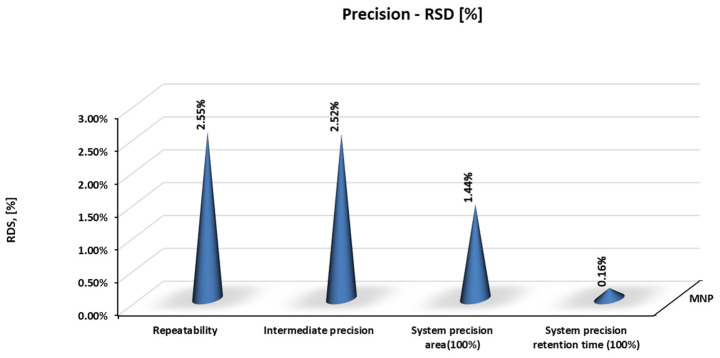
Results of precision for MNP content in 4-component Rifampicin product.

As demonstrated in Figure 4, method precision for MNP content determination was evaluated by repeatability, intermediate precision, and system precision, with satisfactory RSD values below 2.6%.

The LOD and LOQ were calculated both ways from linearity and ratio peak/noise height, the calculations were very similar and are summarized in Table 1. The results for LOD and LOQ were satisfactory and LOQ was (as in acceptance criteria) below 30% of the specification limit. The MRM chromatograms for LOD and LOQ are shown in Figures S13 and S14. The robustness of the LC-MS/MS method was evaluated on the standard solution to determine critical factors that most likely can affect an HPLC separation. The evaluation of method robustness was conducted by applying small variations of chromatographic parameters: column temperature ±5 °C, autosampler temperature: ±5 °C, flow rate: ±0.05 mL/min, and mobile phase composition: ±2% eluent B. The results (Table S2) confirmed the robustness of the method. The symmetry factor for MNP peak was in the acceptance criteria range of 0.8 ≤ As ≤ 1.5. 

Moreover, during validation stability for reference solution at 100% concentration level, LOQ solution, and sample solution of four-component rifampicin product under autosampler conditions were investigated. RSD% (area quotient MNP/MNP-d4) for reference solution at 100% concentration level after 24 in 10 °C was 0.98%, for LOQ solution after 24 h in 10 °C was 5.05%, and for sample solution of four-component rifampicin product after 8 h in 10 °C 4.6%, respectively (Tables S3–S5). The research showed the stability of tested solutions over the time period studied.

### 2.3. Stability and Stress Studies 

**Figure 5:**
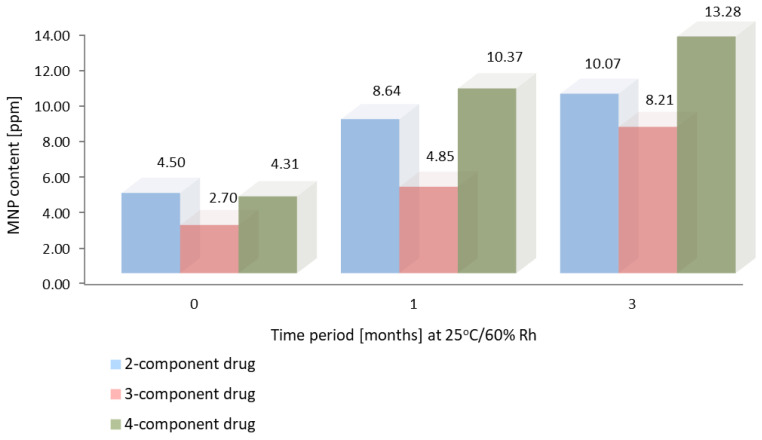
The results of accelerated stability studies (25 °C, 60% RH) for 2, 3, and 4-component Rifampicin products.

**Figure 6:**
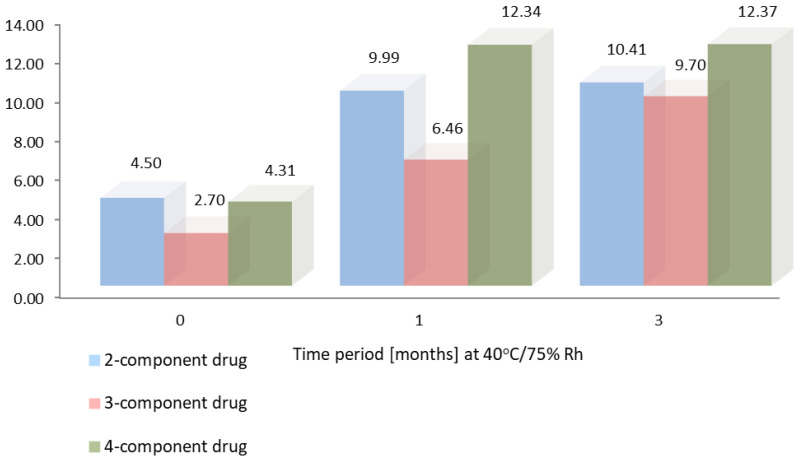
The results of accelerated stability studies (40 °C, 75% RH) for 2, 3, and 4-component Rifampicin products.

Figure 5 and Figure 6 show the results of accelerated stability studies performed according to ICH guidelines after 1 and 3 months of storage at 25 °C, 60% RH, and 40 °C, 75% RH, respectively [33]. The MNP content of the 4-component product with rifampicin increased, as confirmed by previous studies for API (30 days) [13]. 

The results of the forced degradation studies carried out in accordance with ICH guidelines and publications [33,34] are summarized in Table 2 [33,34]. As reported before a rapid decomposition of rifampicin is observed in acid, alkaline, oxidative and thermal (>70 °C) conditions [35,36,37]. In our experiments, a significant increase in MNP concentration was observed in an oxidizing environment, as well as in thermal stress that takes place in an airy atmosphere.

For further identification of the mechanism of MNP formation in rifampicin products, additional experiments were performed investigating the presence of both amines: 1-methyl-piperazine and 1-amino-4-methyl-piperazine (4A1MA), and MNP before and after degradation (Table 3). In samples of both API and the 4-component rifampicin drug, the presence of 1-amino-4-methyl-piperazine, which comes from the synthesis pathway, was detected. Compound 1-methyl-piperazine was not found in the samples tested.

The content of this 1-amino-4-methyl-piperazine increases under acidic conditions due to the fact that rifampicin decomposes to 1-amino-4-methylpiperazine under these conditions, as described in the literature [38]. Next, a hydroxide water addition experiment was performed and oxidation of the amine to MNP was observed (Table 3).

## 3. Discussion

The developed and validated method was used to assess the presence of MNP in rifampicin multicomponent drugs. In the two-component rifampicin drugs, the MNP content ranged from 3.7 to 4.7 ppm, in the three-component drugs from 2.5 to 2.7 ppm, and in the four-component drugs from 3.9 to 7.5 ppm.

Forced degradation studies are essential to establish the stability of the API/drug product, learn about potential toxic degradation products, and also to develop sensitive analytical methods for stability specification. In our study, stress tests (thermal, oxidative, alkaline and acid hydrolysis) were conducted to investigate the changes in the MNP content in a four-component drug product with rifampicin and to confirm possible mechanisms of the formation of this nitrosamine. 

In our experiments, a significant increase in MNP concentration was observed in an oxidizing environment, as well as in thermal stress that takes place in an airy atmosphere. The content of 1-amino-4-methyl-piperazine increases under acidic conditions due to the fact that rifampicin decomposes to 1-amino-4-methylpiperazine under these conditions, as described in the literature [38]. In acid conditions, the main decomposition product of rifampicin is 3-formyl rifampicin SV and 1-amino-4-methyl-piperazine [35,38,39]. Next, under oxidative conditions, the resulting amine is further oxidized to MNP, the content of which increases. The MNP content does not increase in direct proportion to the decrease in amine content, indicating that simultaneous oxidation of the amine and further degradation of rifampicin is observed. These results were also confirmed using the LC-QTOF method, high-resolution mass spectra, and elemental compositions for MNP and 1-amino-4-methylpiperazine are included in Supplementary Material (Table S6 and Figures S15–S18).

**Figure 7:**
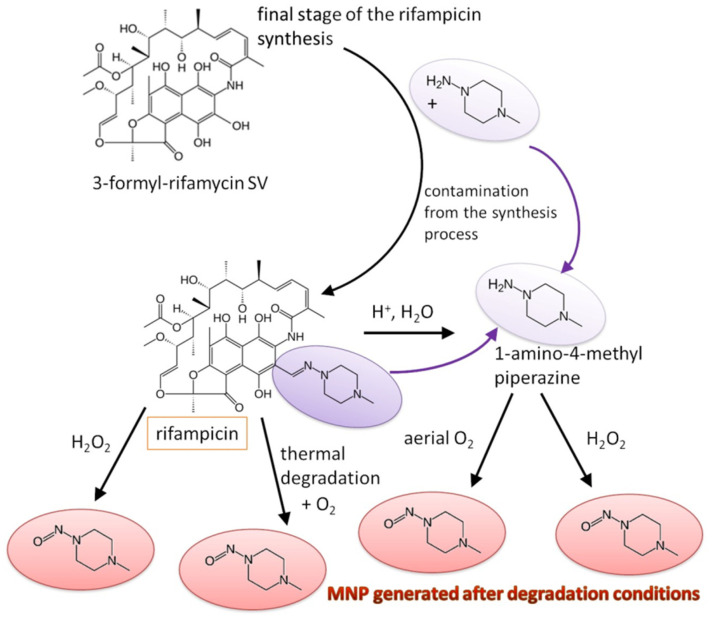
Mechanisms of MNP formation during degradation studies.

Based on this study, we proposed a mechanism for MNP formation during degradation studies, which is shown in Figure 7. 

Considering the process of the formation of nitrosamines into a drug product, especially a multicomponent one, is quite complex and challenging. The literature indicates that three conditions must be met for nitrosamines to be formed: the presence of a nitrosating agent or its precursor (nitrites), the presence by synthesis of a secondary or tertiary amine (which may be a component of the drug substance), and suitable conditions to allow the reaction to take place, such as elevated temperature, acidic conditions [40,41,42]. API degradation is another mechanism that can lead to the formation of nitrosamines. In the case of rifampicin, only a temperature effect on MNP formation has previously been suggested [13]. Our study confirmed the effect of temperature on the MNP content in 2, 3, and 4-component products with rifampicin with access to atmospheric oxygen. With increasing storage temperature, humidity and time period, the MNP content of rifampicin drugs increases. The degradation studies described in this thesis revealed two mechanisms for the formation of MNP: the first through the oxidation of 1-amino-4-methylpiperazine (derived from the synthesis route), and the second through rifampicin degradation. As recommended by the FDA and EMA regulations, the amine content of products containing nitrate by synthesis should be strictly controlled and limited. The case of rifampicin clearly demonstrates that the formation of the nitrosamine MNP is also closely related to the presence of residual amine. 

We propose a degradation mechanism for the formation of MNP by hydrolysis of a hydrazone of rifampicin to a hydrazine derivative, in this case to 1-amino-4-methylpiperazine. The hydrazine derivative can be oxidized to form an MNP impurity. It should be borne in mind that hydrazones are stable and hydrolyze in an acidic medium to hydrazine derivatives, while hydrazine derivatives can oxidize to the corresponding nitrosamine impurity. Thus, we propose a two-step mechanism for rifampicin degradation: hydrolysis of the hydrazone followed by oxidation to MNP. It is worth noting that not all of 1-amino-4-methylpiperazine oxidizes to MNP, but the content of MNP increases significantly when the oxidizing agent is added. The second mechanism we propose is different and explains the formation of MNP at elevated temperatures. This is because it is possible that degradation occurs without the breakdown of the hydrazone. On the other hand, in this case, too, an oxidizing agent is needed, so that oxygen can first attach to the rifampicin structure and perhaps then break the bond giving the MNP impurity, or the bond breaks first and oxygen attaches later. 

The temperature-induced degradation of rifampicin to MNP can also be inhibited if there is no access to oxygen and humidity from the air. We conclude that both oxygen, humidity, and acidic conditions can degrade rifampicin to MNP. Consider the fact that ethambutol hydrochloride added to rifampicin products is a hydrochloride and can be a source of such hydrolysis.

## 4. Materials and Methods

### 4.1. Materials

The standards 1-Methyl-4-nitrosopiperazine and 1-Methyl-4-nitrosopiperazine-d4 were obtained from TRC (North York, ON, Canada). Methanol hypergrade for LC-MS LiChrosolv^®^, hydrogen peroxide 30% (Perhydrol™, Sigma-Aldrich, St. Louis, MO, USA), hydrochloric acid 37% and sodium hydroxide were obtained from Merck (Darmstadt, Germany). LC-MS grade water was purchased from Fisher Scientific (Hampton, NH, USA). Rifampicin and rifampicin finished medical products were acquired from several Marketing authorization holders (MAHs).

### 4.2. Equipment and Methods

The LC-MS/MS analysis was performed using 1290 LC (Agilent, Santa Clara, CA, USA) coupled with a 6460 Agilent triple quadrupole mass spectrometer with a Jetstream electrospray source. For LC analysis Purospher^®^ STAR Phenyl (10 mm × 2.1 mm × 2.0 µm) column from Merck (Darmstadt, Germany) was used. All LC-MS/MS method experimental conditions are presented in Table 4. The MRM ion pair for MNP and MNP-d4 with optimized parameters are listed in Table 5, the MS monitoring time was 2.9 to 5 min.

The key results for stress studies were also confirmed on a MaXis 4G high-resolution, mass-accuracy mass spectrometer (Bruker Daltonic, Bremen, Germany) with a time-of-flight (TOF) analyzer coupled to an Ultimate 3000 ultra-high performance liquid chromatography (UHPLC) (Thermo Fisher Scientific, Dreieich, Germany) to obtain a high-resolution mass spectrum and confirm the chemical formula. HPLC experimental conditions were the same as in the LC-MS/MS method.

### 4.3. Standard Solution Preparation

The MNP-d4 internal standard (IS) solution (1 µg/mL in methanol) was prepared from the internal stock solution MNP-d4 (5000 µg/mL in methanol). The MNP intermediate dilution was prepared by dissolving appropriate amounts of MNP certified reference material to reach 0.1 µg/mL of MNP in MeOH.

### 4.4. Sample Solution Preparation

135 mg of rifampicin four-component product (10 tablets thoroughly grated) was weighed into a 15 mL centrifuge tube and 100 µL of the internal standard solution, 2.4 mL of methanol, 2.35 mL of water and 150 µL 1 M NaOH (neutralization) were added. The mixture was vortex 1 min at 2000 rpm and then centrifuged by 10,000× *g* rpm for 5 min. The supernatant was filtered through a 0.2 µm PTFE Whatman Mini-UniPrep ™ filter (Cytiva, Global Life Sciences Solutions Operations UK Ltd., Little Chalfont, Buckinghamshire, UK).

### 4.5. Blank Preparation

The blank solution was prepared as described in Section 4.4, but without the addition of the sample. 

### 4.6. Validation of the LC-MS/MS Method 

The LC-MS/MS method validation for MNP content determination in rifampicin multi-component products was performed according to ICH guidelines on the following parameters: specificity, linearity, range, accuracy, repeatability, intermediate precision, robustness, stability of solutions, quantitation limit, and detection limit. 

The specificity of the method was examined using blank solution, standard solution at 100% concentration level, sample solution of drug product, sample solution spiked with standard solution of MNP at 100% concentration level, 1-methyl-piperazine solution and 1-amino-4-methyl-piperazine solution (100 ng/mL) (Figures S1–S12). The calibration curve was prepared (from MNP intermediate dilution) at concentrations from LOQ to 280% (0.5–48.6 ng/mL) of the specified limit for MNP with respect to sample preparation due to high MNP content in the sample. The internal standard solution MNP-d4 was added at a concentration of 20 ng/mL. Slope a, intercept b and coefficient of determination R^2^ and standard deviation S_a_, S_b_, S_xy_ were established as parameters characterizing the linearity. The accuracy of the method was evaluated at samples spiked with MNP at 80%, 100% and 120% of specification limit—9 sample solutions (triplicate independent preparations for solutions at each level). The acceptance criteria were: 60–115% and the precision at each concentration should not exceed RSD < 21% according to the Association of Official Analytical Chemists [43].

The precision of the method was determined as repeatable—analysis of six sample solutions with intermediate precision—intra-laboratory variations, i.e., different day and analyst and system precision—six replicate injections of the standard solution. Acceptance criteria for the precision of the method were as follows: RSD should not exceed 21%, and the results should pass the Horwitz statistical test.

LOD and LOQ were calculated in two different ways. One with the Agilent software (B.07.00) using the signal-to-noise (S/N) ratio, where LOQ was defined as S/N ≥ 10 and LOD as S/N ≥ 3 and using linearity parameters: standard deviation of the response (σ) and slope of the curve (S), where LOD = (3.3 × σ/S) and LOQ = (10 × σ/S).

The robustness of the developed method was tested by injecting a standard solution after applying minor changes in chromatographic parameters: column temperature ±5 °C, autosampler temperature ±5 °C, flow rate ±0.05 mL/min and mobile phase composition (±2% of eluent B). The symmetry factor for the MNP peak and the resolution during this study were verified.

The stability of the solutions was evaluated by measuring the peak area quotient of MNP and MNP-d4 for reference solution at 100% concentration level (24 h, 10 °C), LOQ solution (24 h, 10 °C) and sample solution of Rifampicin product (four-component, 8 h, 10 °C). The peak area mean Standard Deviation (SD) and Relative Standard Deviation (RSD%) for each solution were calculated.

### 4.7. Stress Studies 

The forced degradation test solutions (0.1 M NaOH, 0.1 M HCl, 3% H_2_O_2_) were prepared as follows: 135 mg of sample was weighed into a 15 mL centrifuge tube, 2.5 mL of 0.1 M NaOH/0.1 HCl/3% H_2_O_2_ solution was added and stirred for 1 h. The sample was neutralized. Then, 2.4 mL of methanol and 100 µL of the MNP-d4 internal standard solution were added. The tube was capped, vortexed for 1 min at 2000 rpm and the solution was centrifuged at 10,000× *g* rpm for 5 min. The supernatant was filtered through a 0.2 µm PTFE Whatman Mini-UniPrep ™ filter.

The thermal stress study at 80 °C, 24 h was prepared as described in Section 4.4, but the sample was first placed on a Petri dish in an oven for 24 h at 80 °C.

## 5. Conclusions

The LC-MS/MS method was developed for the determination of MNP—a genotoxic nitrosamine impurity in two-, three-, and four-component drug products (rifampicin/isoniazid/pyrazinamide/ethambutol hydrochloride). In the case of the four-component product, an effect of the matrix/solvent was observed, which required the development of appropriate sample preparation for analyte extraction. The method of quantitative determination of MNP was validated to ensure its appropriate use with a Limit Of Detection (LOD) of 0.15 ppm in two- and three- and four-component medicines. The developed method was successfully applied to the analysis of rifampicin products from various manufacturers, providing the first detailed insight into MNP contamination. The study has demonstrated various levels and sources of MNP: as an impurity from the synthesis pathway and as a degradation product of rifampicin. MNP formation in medical products with rifampicin was found to be time, temperature, and degradation environment (0.1 M HCl, 3% H_2_O_2_) dependent. Additional degradation experiments that can explain the origin of MNP impurity were conducted and the mechanism of this impurity formation was discussed. It was emphasized that the content of 1-amino-4-methyl-piperazine should be reduced and strictly controlled due to the possibility of its oxidation to MNP. A much more serious mechanism appears to be that of direct oxidation of rifampicin and its degradation to MNP. Work to elucidate this mechanism continues in our laboratory.

The developed method is intended to provide implementers with insight into where MNP contamination is coming from, and how the identification and quantification of MNP in multicomponent rifampicin products may impact the safety of medical products.

## Figures and Tables

**Figure 2:**
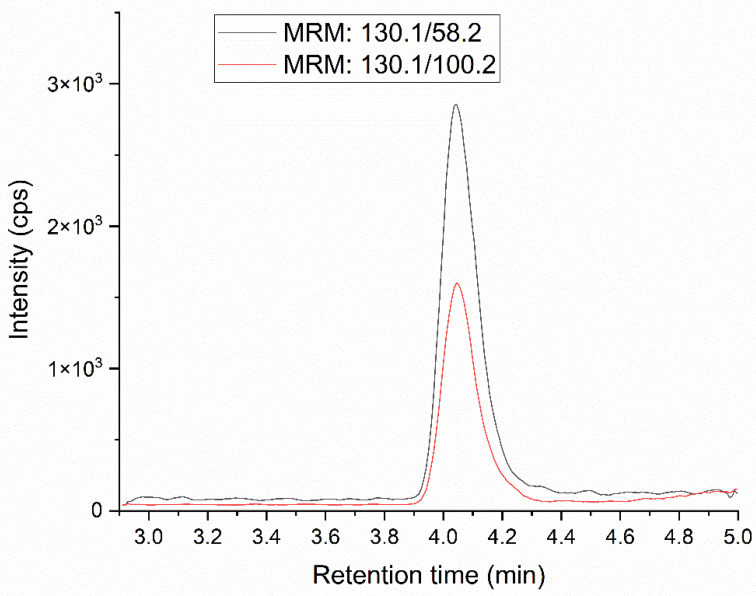
MRM chromatogram of standard solution at 100% concentration level for MNP (130.1 > 100.2 red curve, 130.1 > 58.2 black curve).

**Figure 3:**
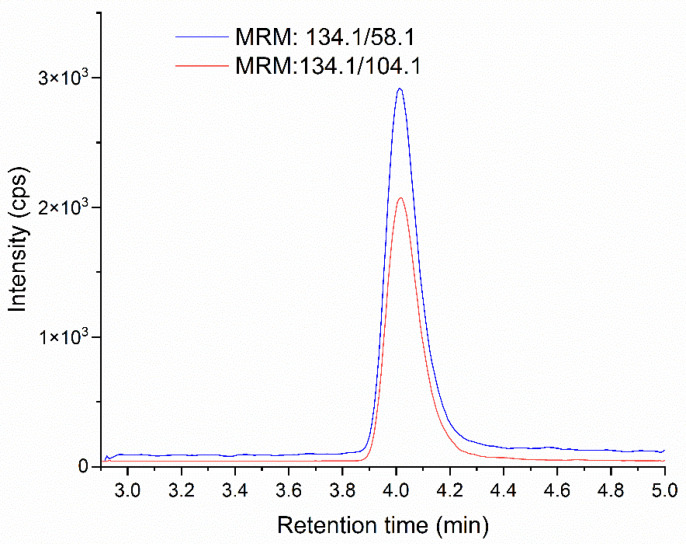
MRM chromatogram of standard solution at 100% concentration level for MNP-d4 (134.1 > 58.1 blue curve, 134.1 > 104.1 red curve).

**Table 1:** Validation results for LC-MS/MS method.

Acceptance Criteria	Results for MNP
Range: LOQ-120% [ng/mL]	0.51–48.62
Linearity	
R^2^ ≥ 0.990	0.9997
a	0.0499
b	0.0096
S_a_	0.0003
S_b_	0.0074
S_xy_	0.0152
Accuracy	
Recovery 60–115%	100.38
RSD ≤ 21%	8.17
LOD [ng/mL]	0.51
LOQ [ng/mL]	1.52

**Table 2:** The results of stress studies for four component Rifampicin product.

Degradation Conditions	MNP Content [ppm]
Control sample (before degradation)	7.07
Alkaline hydrolysis (0.1 M NaOH, 1 h, RT)	7.26
Oxidative degradation (3% H_2_O_2_, 1 h, RT)	2920
Thermal stress (80 °C, 24 h, RT)	8.62

**Table 3:** MNP and 4A1MA content in rifampicin and 4-component rifampicin product after degradation studies.

Stress Conditions	1-Amino-4-Methyl-Piperazine [ppm]	MNP [ppm]
API		
control	0.841	3.146
0.1 HCl, 1 h, 25 °C	3.020	3.648
0.1 HCl, 1 h, 25 °C + 3% H_2_O_2_	2.821	2601
4-component rifampicin product		
control	10.186	3.871
0.1 HCl, 1 h, 25 °C	20.922	3.616
0.1 HCl, 1 h, 25 °C + 3% H_2_O_2_	10.494	654

**Table 4:** LC-MS/MS method experimental conditions.

LC Parameters			
Mobile phase A	10 mM ammoniu formate pH = 9		
Mobile phase B	Methanol		
Gradient elution program:	Time, min	Eluent A, %	Eluent B, %
	0	90	10
	2.0	90	10
	5.0	5	95
	7.0	5	95
	7.2	90	10
	10.0	90	10
Column temperature	35 °C		
Autosampler temperature	10 °C		
Flow rate	0.3 mL/min		
Injection volume	5 µL		
MS parameters			
Gas temperature	350 °C		
Gas flow	11 L/min		
Nebulizer	50 psi		
Sheath gas temperature	350 °C		
Sheath gas flow	12 L/min		
Capillary	3000 V		
Nozzle Voltage	500 V		
Fragmentor	40		

**Table 5:** LC-MS/MS MRM transitions list.

Analyte Name	RT (min)	Precursor Ion	Fragmentor (V)	Product Ion 1	CE 1 (V)	Product Ion 2	CE 2 (V)
MNP	3.985	130.1	40	100.2	10	58.2	18
MNP-d4	3.944	134.1	70	104.1	8	58.1	24

## Data Availability

Not applicable.

## Supplementary Materials

The following supporting information can be downloaded at: https://www.mdpi.com/article/10.3390/molecules28217405/s1.
