# Supplementary material for: Development and Validation of LC-MS/MS Method for the Determination of 1-Methyl-4-Nitrosopiperazine (MNP) in Multicomponent Products with Rifampicin—Analytical Challenges and Degradation Studies
Source: Molecules. 2023 Nov 3;28(21):7405. doi: 10.3390/molecules28217405 (PMC10648194; doi:10.3390/molecules28217405)
Supplement: Supplementary file 1 [file molecules-28-07405-s001.zip › molecules-2688021-supplementary.pdf]

Supplementary Material

Development and validation of a universal LC-MS/MS method for the determination of 1-methyl-4-nitrosopiperazine (MNP) in multicomponent products with rifampicin - challenges and degradation studies

[1] Spectrometric Methods Department, National Medicines Institute, 30/34 Chełmska, 00-725 Warsaw, Poland; anna.witkowska@wum.edu.pl (A.B.W.); a.wolczynska@nil.gov.pl (A.W.); a.lis@nil.gov.pl (A.L.-C.)

[2] Department of Drug Chemistry, Doctoral School, Medical University of Warsaw, 61 Żwirki i Wigury, 02-091 Warsaw, Poland
* Correspondence: e.stolarczyk@nil.gov.pl

Table S1. MNP content in the 3-component product - methods 1 and 2 comaprison.

Method	MNP content [ppm]
**Method 1 - Extraction with up to 5 mL of methanol**	2.69
**Method 2- extraction - water with the addition of NaOH /methanol**	2.70

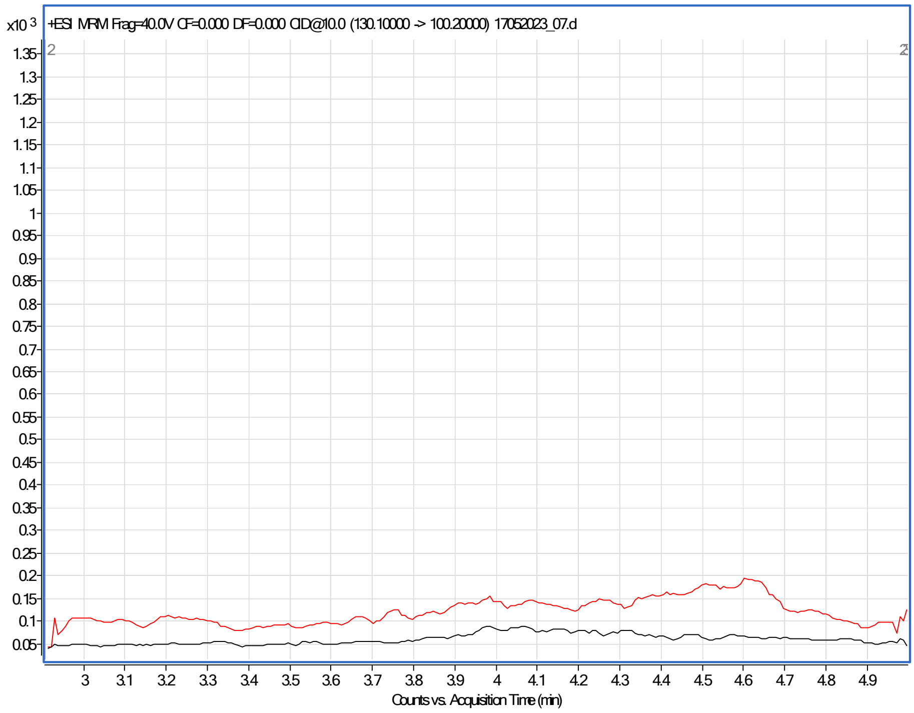

Figure S1. MRM chromatogram of blank for MNP (130.1 > 58.2 red curve, 130.1 > 100.2 black curve).

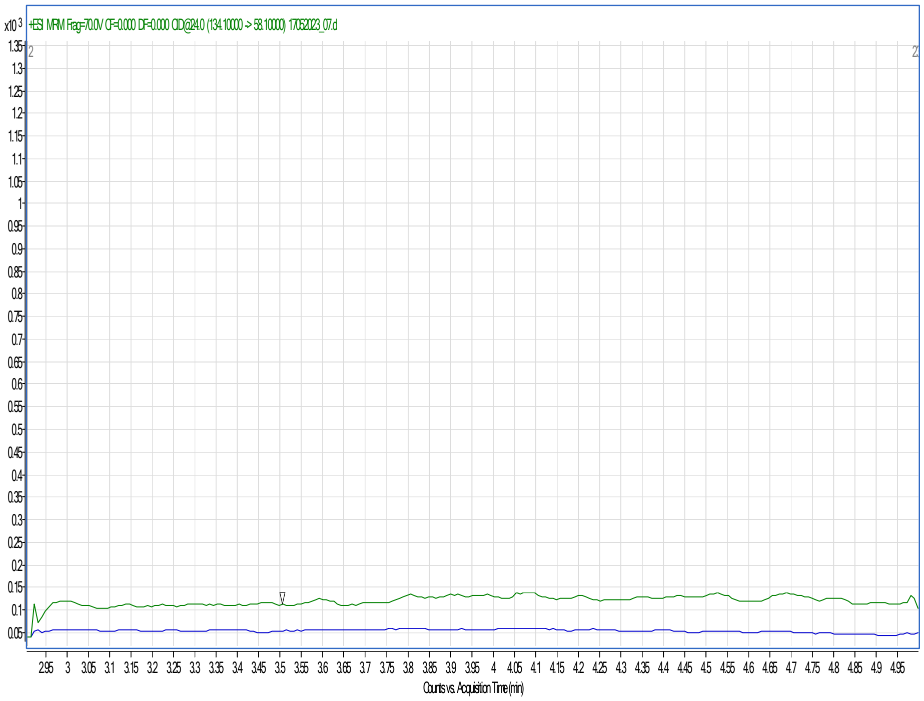

Figure S2. MRM chromatogram of blank for MNP-d4 (134.1 > 58.1 green curve, 134.1 > 104.1 blue curve).

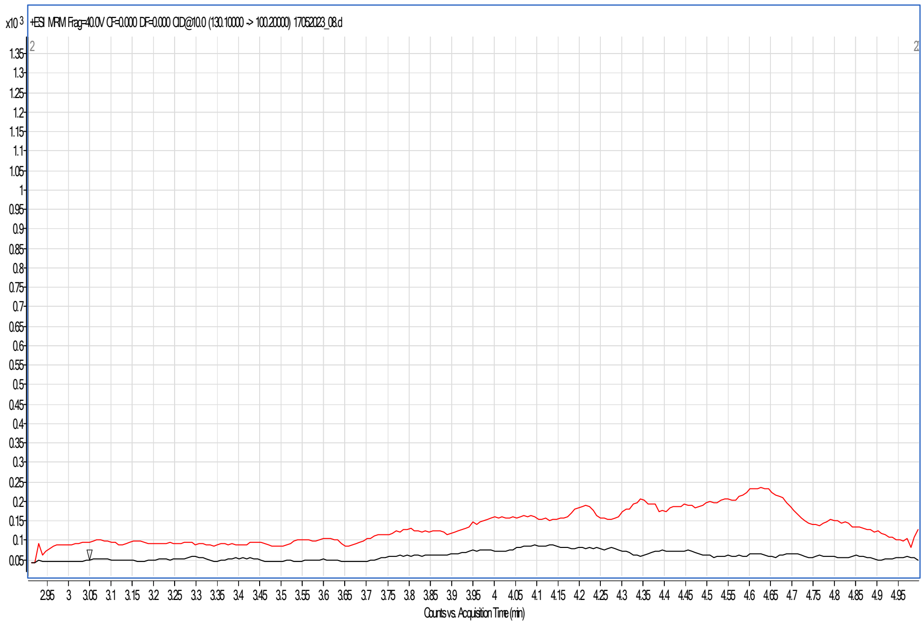

Figure S3. MRM chromatogram of blank solution spiked with the internal standard solution MNP-d4 for MNP (130.1 > 58.2 red curve,130.1 > 100.2 black curve).

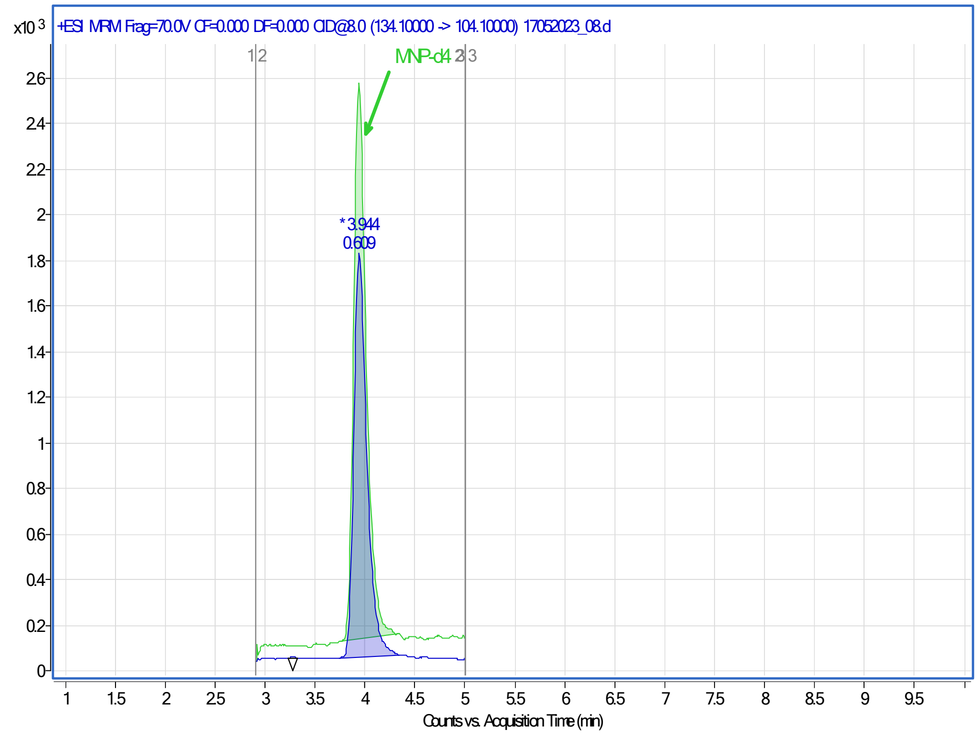

Figure S4. MRM chromatogram of blank solution spiked with the internal standard solution MNP-d4: MNP-d4 (134.1 > 58.1 green curve, 134.1 > 104.1 blue curve).

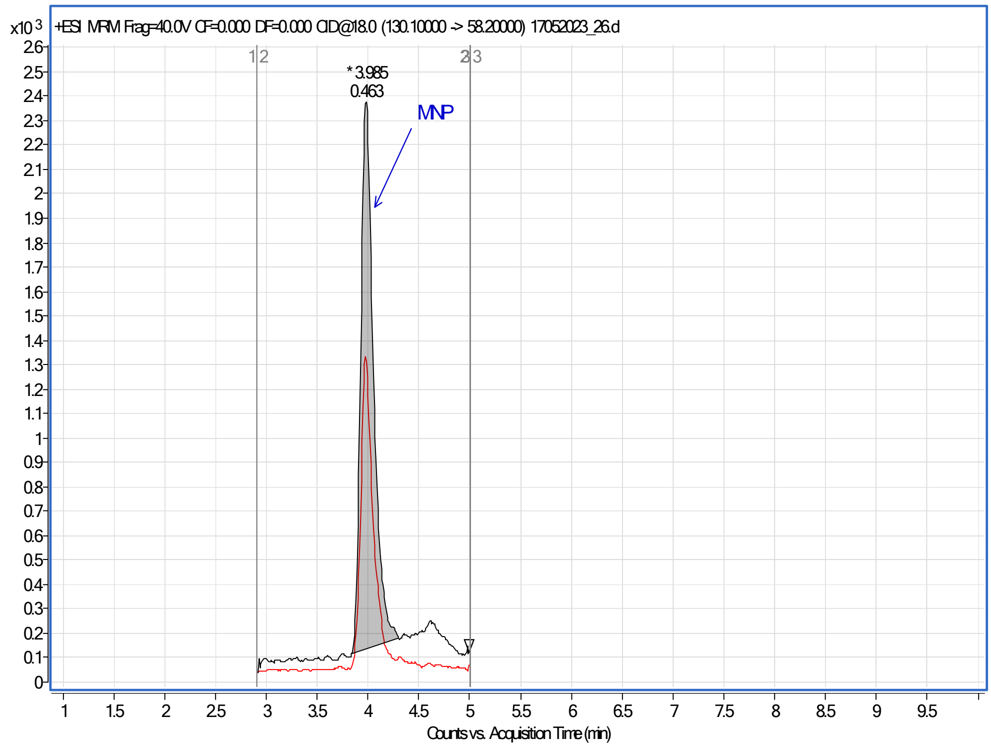

Figure S5. MRM chromatogram of standard solution of at 100% concentration level for MNP (130.1 > 100.2 red curve, 130.1 > 58.2 black curve).

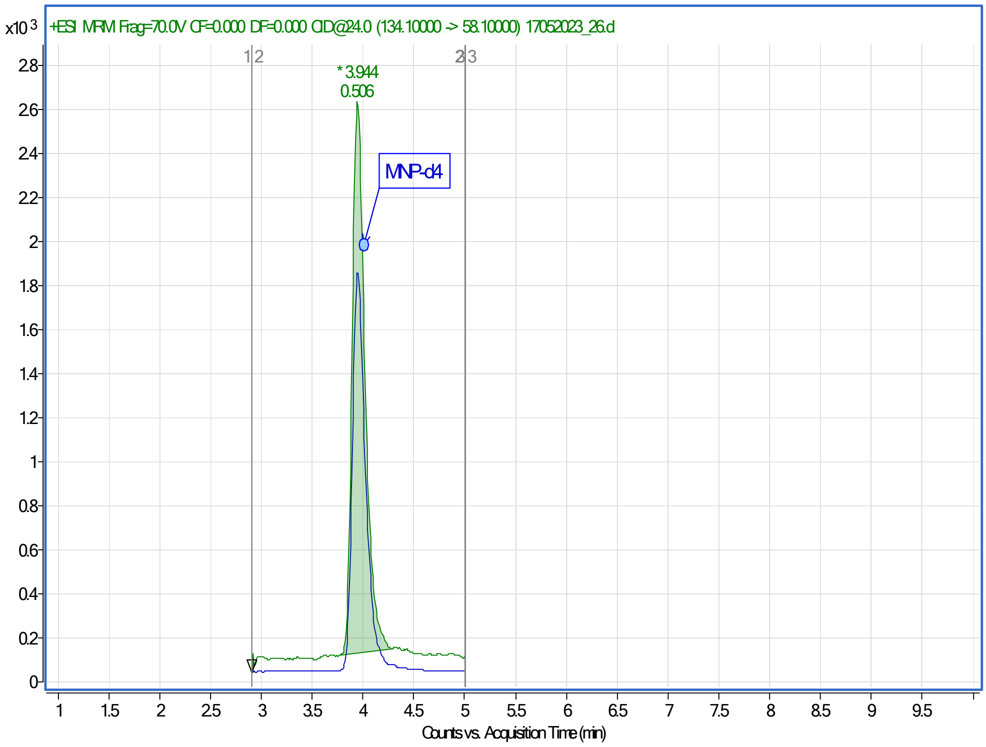

Figure S6. MRM chromatogram of standard solution of at 100% concentration level for MNP-d4 (134.1 > 58.1 green curve, 134.1 > 104.1 blue curve).

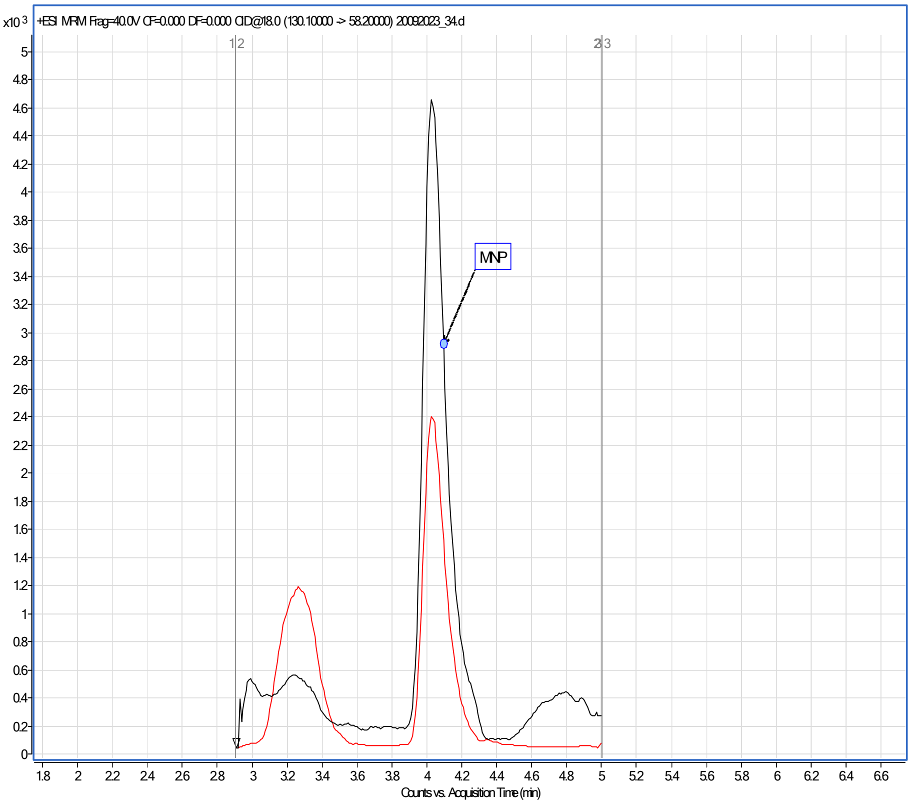

Figure S7. MRM chromatogram of sample solution of drug product (Rifampicin product four-component) for MNP (130.1 > 100.2 red curve, 130.1 > 58.2 black curve).

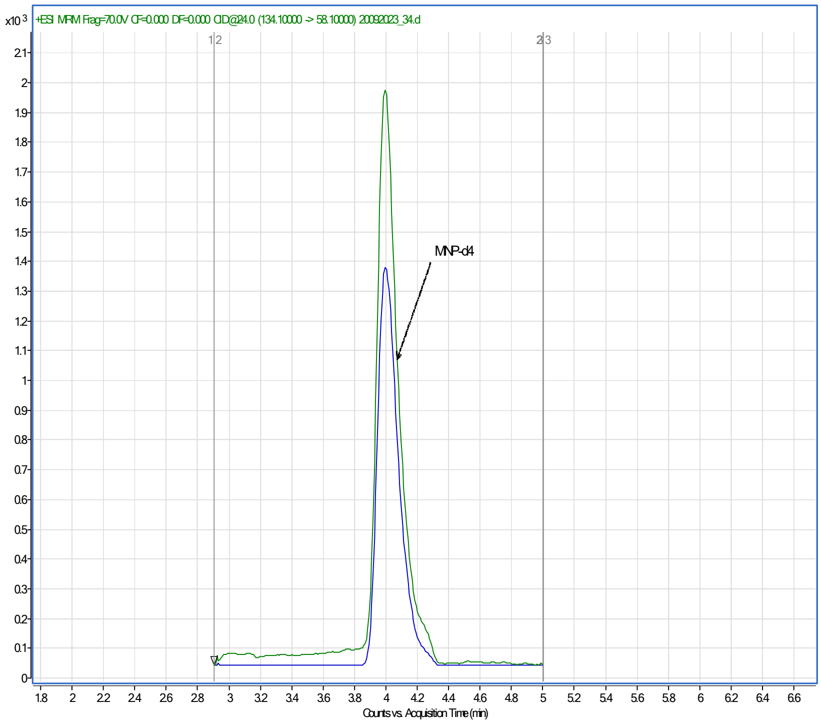

Figure S8. MRM chromatogram of sample solution of drug product (Rifampicin product four-component) for MNP-d4 (134.1 > 58.1 green curve, 134.1 > 104.1 blue curve).

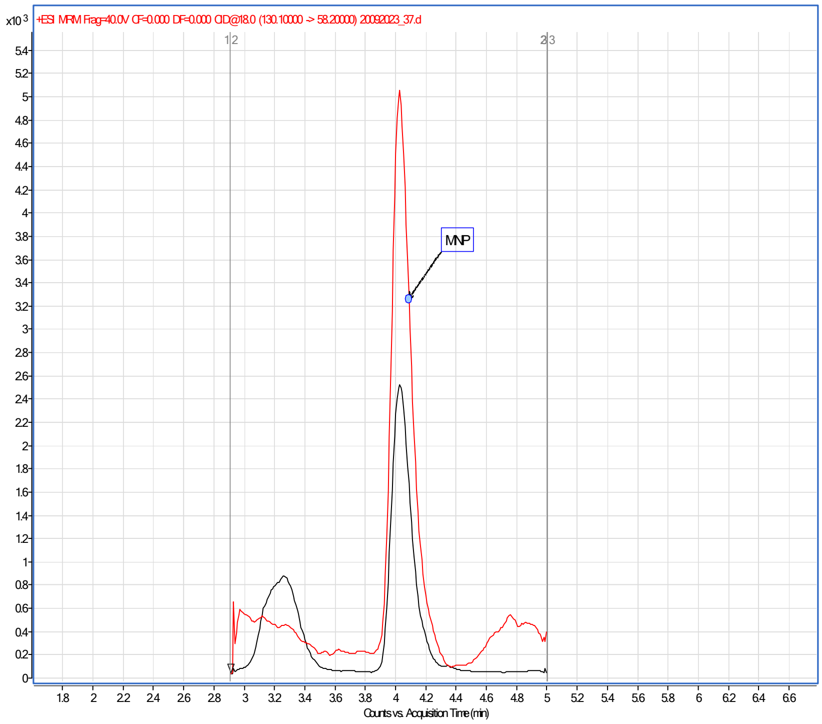

Figure S9. MRM chromatogram of reference solution (sample solution spiked with standard solution of MNP at concentration level of 17.22 ng/mL (5 ppm in respect to sample preparation) for MNP (130.1 > 58.2 red curve,130.1 > 100.2 black curve).

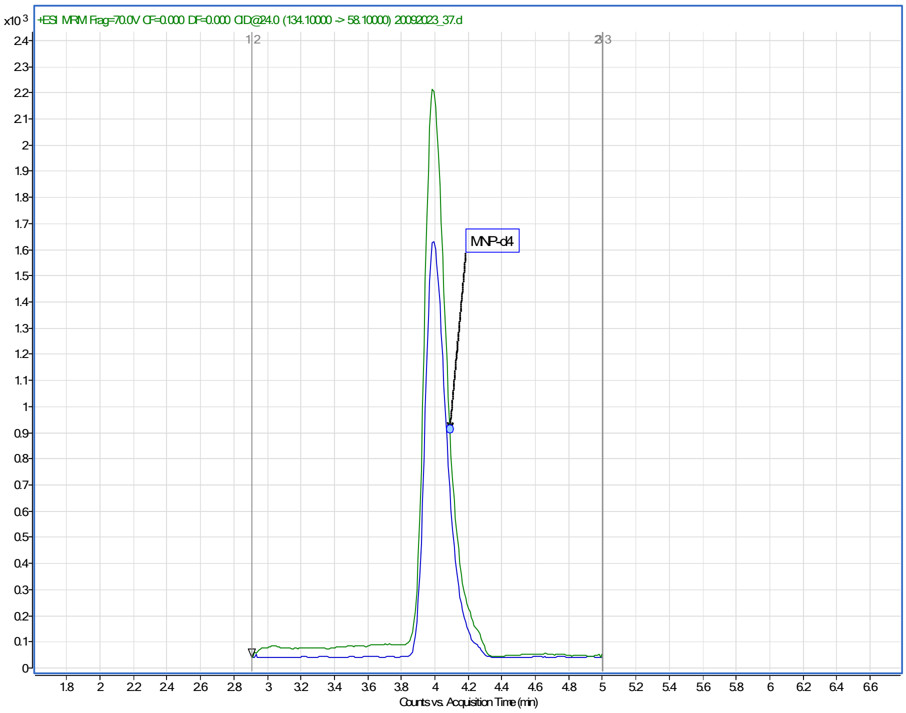

Figure S10. MRM chromatogram of reference solution (sample solution spiked with a standard solution of MNP at the concentration level of 17.22 ng/mL (5 ppm in respect to sample preparation) for MNP-d4 (134.1 > 58.1 green curve, 134.1 > 58.1 blue curve).

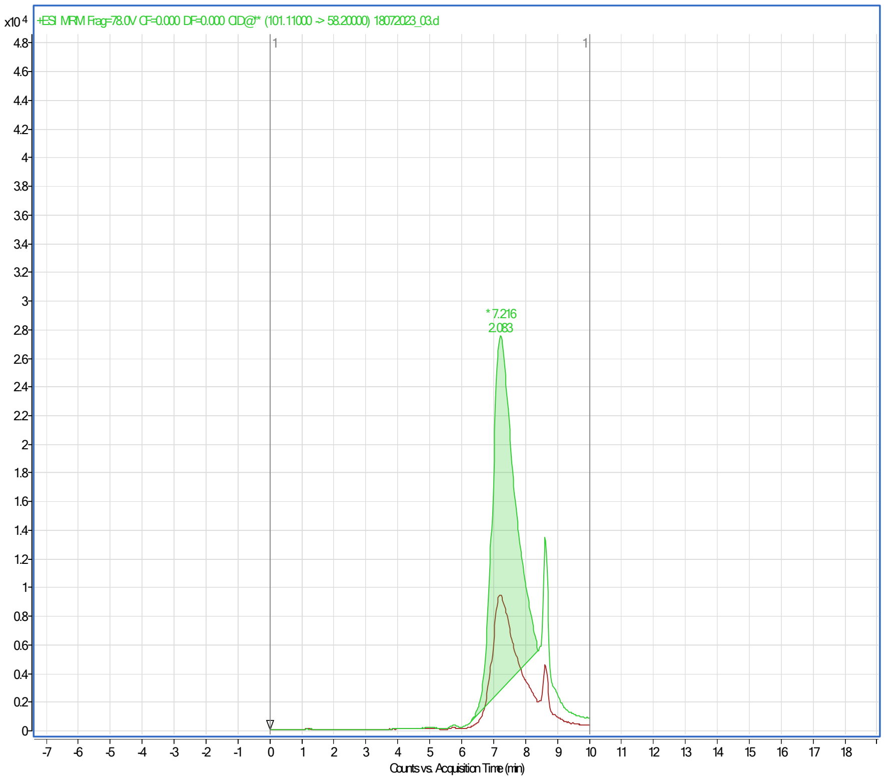

Figure S11. MRM chromatogram of 1-methyl-piperazine solution (101.1 > 58.2 green curve, 101.1 > 44.2 brown curve).

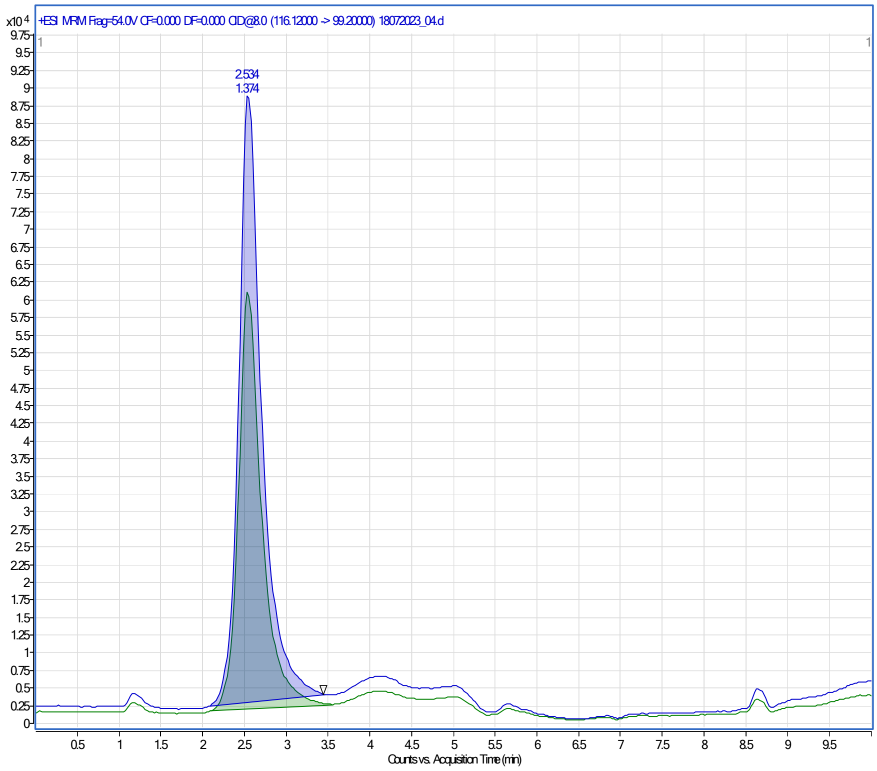

Figure S12. MRM chromatogram of 1-amino-4methyl-piperazine solution (116.12 > 99.2 blue curve, 116.12 > 56.1 green curve).

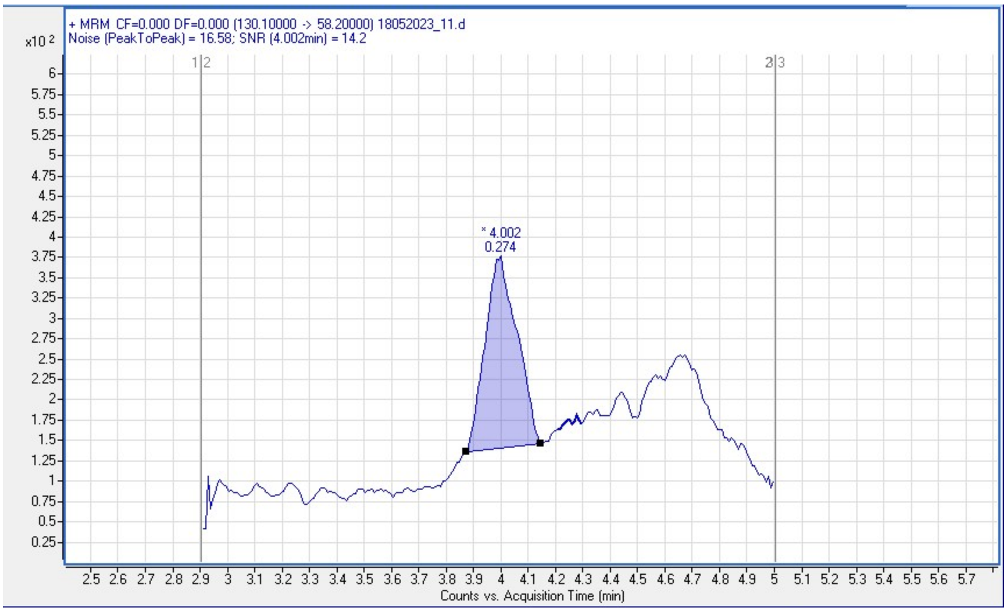

Figure S13. Chromatogram of LOQ solution of MNP 1.52 ppb.

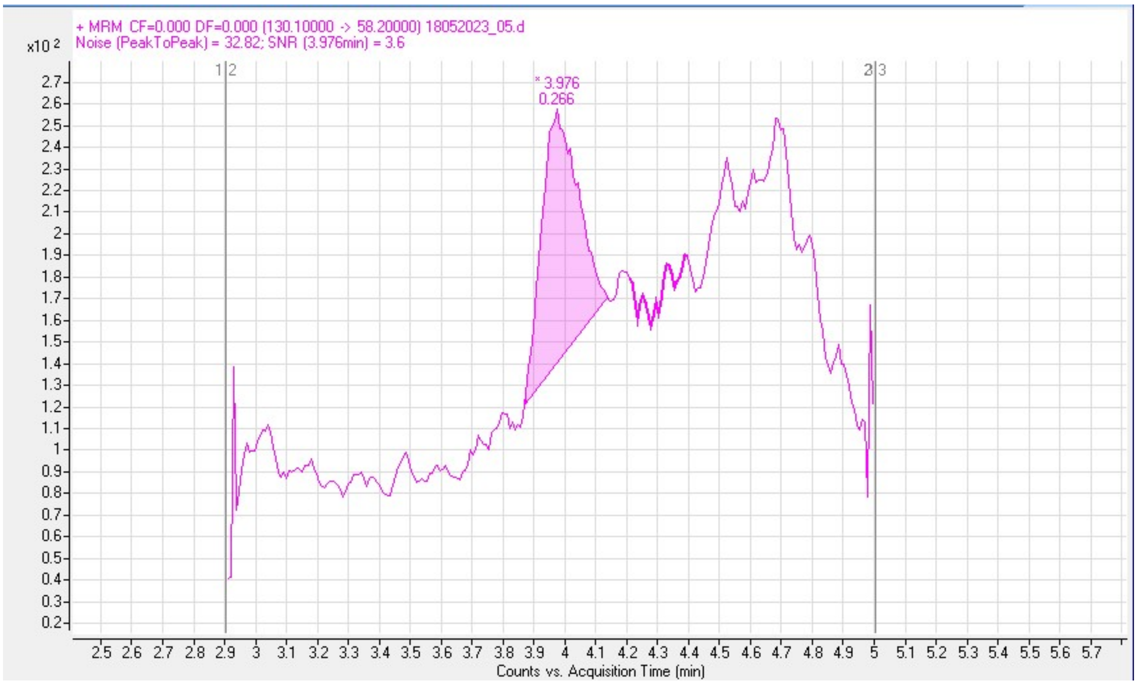

Figure S14. Chromatogram of LOD solution of MNP 0.51 ppb.

Table S2. Robustness of the method. Data for the standard solution of MNP.

**Chromatographic parameters**		**Initial conditions**	**Column temperature.°C**		**Flow rate. mL/min**		**Concentration of eluent B in the mobile phase**		**Autosampler temperature. °C**	
			**30**	**40**	**0.25**	**0.35**	**-2% B**	**+2% B**	**5**	**15**
**MNP**	**t_R. min**	3.986	4.098	3.878	4.510	3.566	4.021	3.969	3.986	3.986
	**A_s**	1.4	1.4	1.4	1.4	1.4	1.3	1.5	1.4	1.3
	**R_s**	34.6	39.6	37.7	40.6	32.6	38.2	36.9	35.5	36.8
**MNP-d4**	**t_R. min**	3.954	4.065	3.842	4.477	3.508	3.98	3.928	3.954	3.954
	**A_s**	1.4	1.4	1.4	1.4	1.4	1.4	1.4	1.4	1.4

Table S3. Results of examined reference solution with MNP at 100% concentration level.

Time	Peak area MNP	Peak area MNP-d4	Area MNP/Area MNP-d4 (Q)
0 h	19737	22144	0.891
0 h	19566	22213	0.881
8h	20916	23626	0.885
8h	20965	23426	0.895
24 h	20936	23666	0.885
24 h	20096	23094	0.870
		Mean	0.885
		SD	0.009
		RSD%	0.978

Table S4. Results of examined LOQ solution.

Time	Peak area MNP	Peak area MNP-d4	Area MNP/Area MNP-d4 (Q)
0 h	1988	0.090	0.090
0 h	1936	0.086	0.086
24 h	1935	0.082	0.082
24 h	1901	0.080	0.080
		Mean	0.085
		SD	0.004
		RSD%	5.048

Table S5. Results of sample solution.

Time	Peak area MNP	Peak area MNP-d4	Area MNP/Area MNP-d4(Q)
0 h	8274	10287	0.804
0 h	7241	8938	0.810
4 h	7796	9735	0.801
8 h	7426	8441	0.880
		Mean	0.824
		SD	0.038
		RSD%	4.556

Table S6. MS summary results for MNP and 1-amino-4-methylpiperazine.

Compoud name	RT, min	Precursor ion, m/z $[M+H]^+$	Theoretical values, m/z	Predicted formula	Err, ppm	mSigma	Product ion (rel.int., %)
1-amino-4-methylpiperazine	3.1	116.1185	116.1182	$C_5H_{13}N_3$	-2.2	15.7	99.0916 (100.00%) 84.0694 (7.25%) 70.0694 (5.21%) 56.0502 (11.24%)
N-methyl_N′-nitrosopiperazine	4.4	130.0977	130.0975	$C_5H_{12}N_3O$	-4.6	17.5	128.0800 (65.02%) 100.0997 (100.00%) 58.0651 (11.22%)

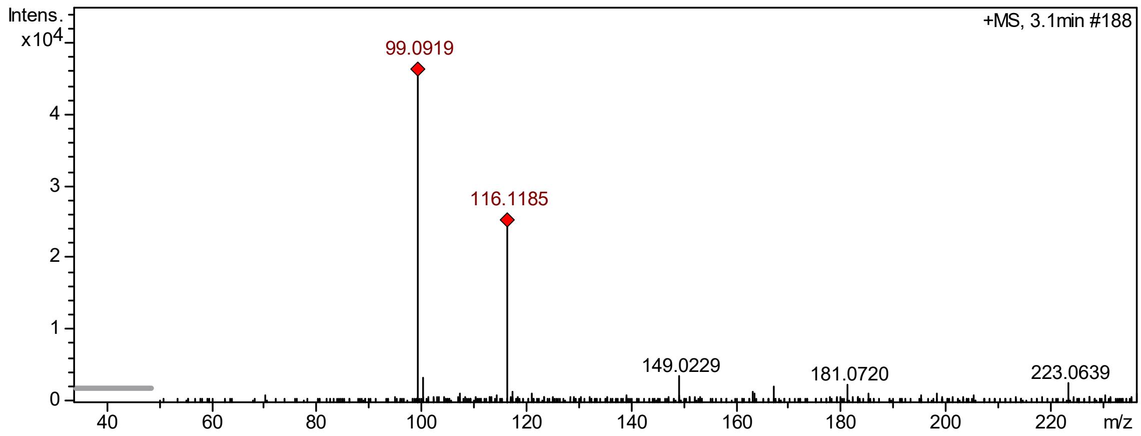

Figure S15. MS spectrum of 1-amino-4-methylpiperazine.

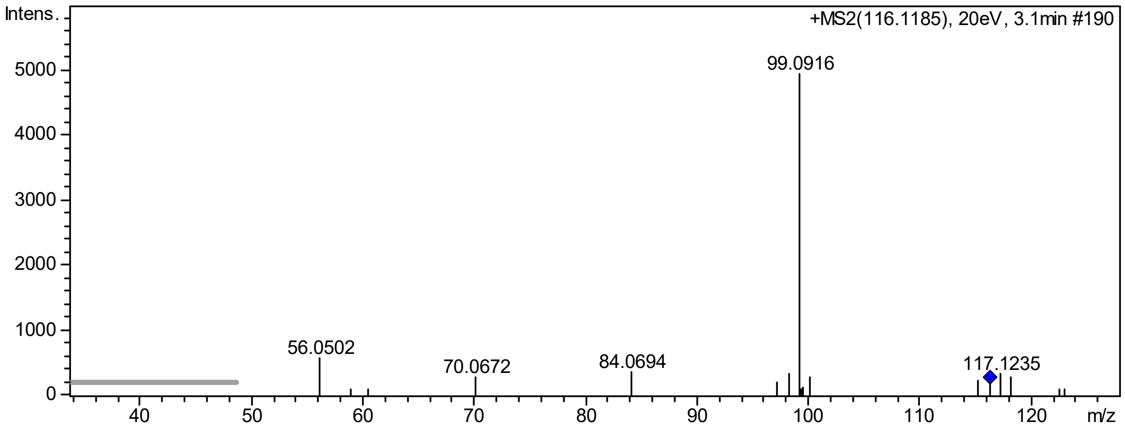

Figure S16. MS/MS spectrum of 1-amino-4-methylpiperazine.

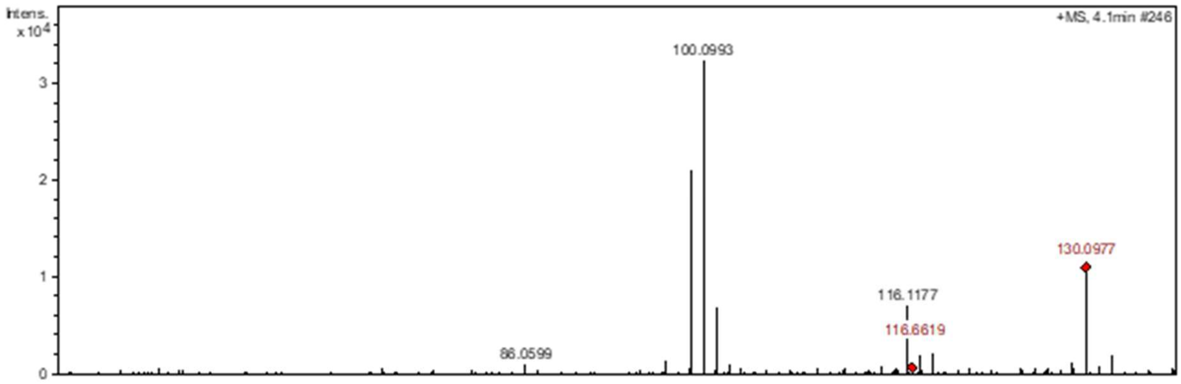

Figure S17. MS spectrum of MNP.

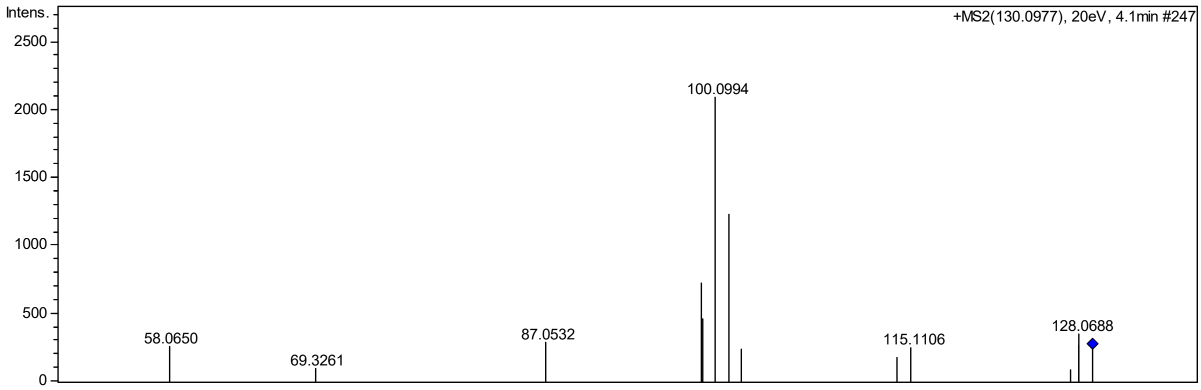

Figure S18. MS/MS spectrum of MNP.
